# Normal Histology

**Published:** 1900-09

**Authors:** 


Normal Histology. By Edward K. Dunham, M. D., Professor of General Pathology, Bacteriology and Hygiene in the University and Bellevue Hospital Medical College, New York. New (2d) edition. In one very handsome 8vo volume of 319 pages, with 244 illustrations. Philadelphia and New York: Lea Brothers & Co., Publishers. 1900. [Price, cloth, $2.50 net.]

The first edition of Dunham's histology was reviewed in the April, 1899, number of this journal, page 717, and the opinion expressed that it would prove highly successful, because the author covered the ground so thoroughly and concisely and stamped his personality on every page of the book. In less than two years a new edition is called for, which gives the author opportunity for making several changes, most important of which is the separating of the work into two volumes, one on normal histology, and the other on pathological histology.

In that on normal histology, which is fresh from the press, he has added such material as has been developed in the brief period since the original publication of his book, and he has likewise appended his concise and complete chapter on technique, so that the book answers every demand of student or physician as a text or laboratory manual. The companion volume on pathological histology is actively preparing. For the advantage of those students whose professors cover both normal and pathological histology in their courses, the original book presenting the whole cognate subject in a single pair of covers will continue to be published.

The volume on normal histology has been rearranged and enlarged so as to cover the field somewhat more in detail than in the first edition and the popularity gained by the former volume cannot fail but fall to the new volume.

Professor Dunham has shown much tact and good judgment in the selection of illustrations as all are beautiful in execution and clear in detail. The volume on pathological histology will be anxiously expected. W. C. K.

